# A case report of missed femoral neck stress fracture

**DOI:** 10.1051/sicotj/2015002

**Published:** 2015-03-06

**Authors:** Oruaro Adebayo Onibere, Hari Kovilazhikathu Sugathan

**Affiliations:** 1 NHS Grampian Aberdeen UK; 2 South Tyneside District Hospital South Shields UK

**Keywords:** Femoral neck stress fracture, Stress fracture, Magnetic Resonance Imaging (MRI)

## Abstract

Femoral neck stress fracture (FNSF) is an uncommon but potentially serious orthopaedic problem. This is a case report on missed femoral neck stress fracture in a 62-year-old female who was initially treated as early-onset coxarthrosis. She later presented to us with a displaced intra-capsular neck of left femur fracture and underwent total hip replacement. This case illustrates that causes other than osteoarthritis should be taken into consideration in patients presenting with anterior hip pain where symptoms are disproportionate to clinical and radiological findings. More advanced investigations such as MRI scan or regular follow up with plain radiographs should be performed. A delay in diagnosis can lead to secondary displacement of the femoral neck stress fracture.

## Introduction

Stress fractures of the femoral neck are uncommon injuries. In general these injuries are seen in two distinct populations: (a) young, healthy, active individuals such as recreational runners, endurance athletes, or military recruits; and (b) the elderly who have osteoporosis. Stress fractures can be classified either fatigue or insufficiency fractures and result from untoward cyclic loading or impaired bone quality [5]. Hence they might be missed in other groups of patients that present with anterior hip pain without history of significant trauma [2]. However it should be taken in consideration as a differential diagnosis because any delay in treatment may lead to potentially serious complications associated with secondary displacement [3]. We present a case report of missed FNSF in a 61-year-old female who was initially treated as early-onset coxarthrosis with intra-articular steroid injection.

## Case report

A 61-year-old previously active, fit and healthy medical secretary presented to our Hospital’s Accident and Emergency Department with severe left hip pain and inability to weight bear for the past 5 days. Her acute symptoms started while she was doing her shopping in a supermarket. She was pushing the shopping trolley and her left leg suddenly gave way, but she did not have a fall. She was unable to bear weight since then. She telephoned National Health Service Direct immediately, but was advised to wait at home and take analgesics. Since her symptoms did not improve her husband brought her to our hospital.

She had been treated by her General Practitioner for early-onset osteoarthritis of the same hip for the past 6 months without regular radiographic follow up. Six weeks prior to the incident she had two local anaesthetic and steroid injections to the hip, given by the physiotherapist. The first injection was intra-articular and the second injection was given to the greater trochanteric area. She has past medical history of hypertension, hiatus hernia and hypothyroidism. She has been on atenolol, lansoprazole, thyroxine and aspirin. She has never been diagnosed with osteoporosis and hence was not on treatment for that.

**Figure 1.**
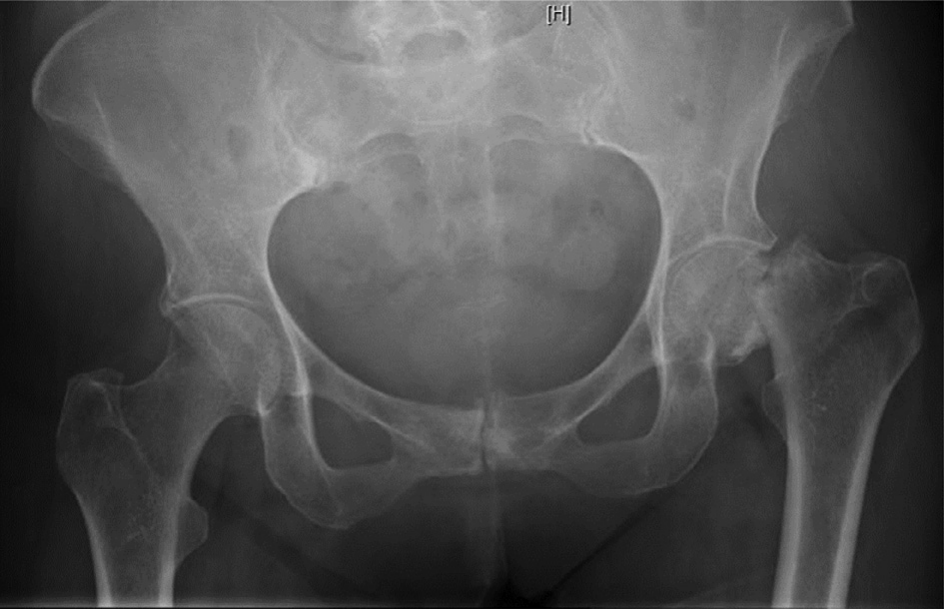
Left hip femoral neck stress fracture.

On examination the leg was shortened and externally rotated. She was unable to do “Straight Leg Raise” or any other active movement of the left hip. Radiographs revealed completely displaced left sub-capital neck of femur fracture and signs of chronic pubic syndesmositis. The appearance suggests that the fracture could have been at least few days old with signs of resorption and sclerosis of the fracture ends Figure 1.

**Figure 2.**
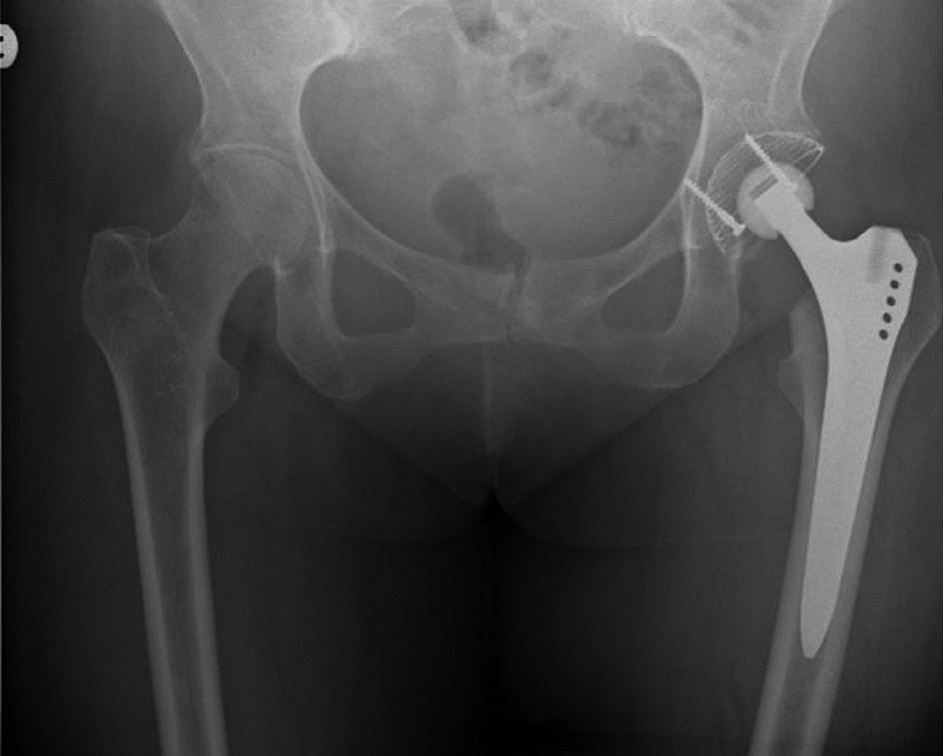
Left total hip replacement.

Patient underwent uncemented left Total Hip Replacement. There was chalky calcified material inside the hip joint suggestive of previous steroid injections. Histopathology also confirmed the fracture to be old with no fresh changes of recent fracture Figure 2.

## Discussion

The incidence of femoral neck stress fractures (FNSF) is varied with a high incidence seen in military recruits of about 3.2%–31%. Femoral neck stress fracture represents 8% of stress fracture are associated with a high morbidity [8].

FNSF represent 3.5%–8% of stress fractures in military recruits; potentially resulting in medical discharge and/or complications.

Femoral neck stress fracture is a rare cause of anterior hip pain and hence diagnosis could be significantly delayed. This can lead to fracture displacement. Patients are often being treated for muscle and tendon strains or early-onset coxarthrosis [2]. Clinical symptoms of FNSF are subtle and non-specific, including painful and possibly limited internal rotation of the hip as well as limping and night pain in more severe cases [2]. Initial plain radiographs are usually inconclusive [1]. Hence high index of clinical suspicion must be maintained with regard to all patients presenting with anterior hip pain without any significant trauma, especially when presenting symptoms are out of proportion to the radiological picture of degenerative changes. As the treatment aims mainly to reduce the pain and sustain patients’ activity level, this may lead to fracture displacement and following complications. Our case illustrates this diagnostic dilemma.

The role of appropriate investigation cannot be over emphasized. Initial plain radiographs of the hip are appropriate. This may reveal obvious neck of femur fractures or displaced femoral neck stress fractures. In the case of elderly patients, it can rule out significant osteoarthritis. Magnetic resonance imaging (MRI) scan and scintigraphy are very useful and important in making a diagnosis of femoral neck stress fracture [5]. This should be done as an emergency, as early treatment reduces and in some cases prevents complications [7].

Due to lack of radiological follow up we agree that it is impossible to confirm that our case was definitely a stress fracture. The clinical, radiological and histopathological data were suggestive of stress fracture. The fracture process could have started at least few days or even weeks before the acute clinical presentation. The patient has been treated by the General Practitioner for early-onset coxarthrosis with in-conclusive evidence and investigations. Radiographs did not confirm the diagnosis of osteoarthritis and later histopathological examination has even excluded it. We believe that intra-articular steroid injection administered 6 weeks prior to the incident gave the patient enough pain relief to sustain her usual activity level, which eventually led to secondary displacement of the fracture.

Single intra-articular steroid injection could not have caused local osteopenia. Some of the complications of FNSF include non-union and osteonecrosis. The incidence of which is increased in displaced FNSF hence the need for early diagnosis and treatment [6]. The diagnosis was missed in our case and the patient required a cemented total hip replacement which would have been a less than ideal outcome in a young patient.

## Conclusions

The learning point with our case report is that all cases of anterior hip pain are not due to early onset osteoarthritis. Other differential diagnosis including stress fracture and avascular necrosis should be considered in patients with minimal radiological evidence of osteoarthritis. Our patient had severe hip pain out of proportion to the clinical and radiological findings. Hence that should have been further investigated with MRI scans or further radiographic follow up or referral to an orthopaedic clinic. All the risk factors of FNSF such as, bone mineral density and patient activity level should be properly assessed. Advanced imaging which induce MRI scan or bone scintigraphy are crucial, as plain radiography is inconclusive at early stages [4]. The most important factor however is regular follow up [3] with regular radiographic review [4] and awareness of this rare cause of anterior hip pain. Any delay in that process may lead to serious complications associated with secondary displacement of the fracture.

## Conflict of interest

Dr. Onibere has nothing to disclose. Dr. Sugathan has nothing to disclose.
